# Supplementary material for: Single-cell analysis of the ventricular-subventricular zone reveals signatures of dorsal and ventral adult neurogenesis
Source: eLife. 2021 Jul 14;10:e67436. doi: 10.7554/eLife.67436 (PMC8443251; doi:10.7554/eLife.67436)
Supplement: Transparent reporting form [file elife-67436-transrepform.docx]

***eLife’s* transparent reporting form**

We encourage authors to provide detailed information *within their submission* to facilitate the interpretation and replication of experiments. Authors can upload supporting documentation to indicate the use of appropriate reporting guidelines for health-related research (see [EQUATOR Network](http://www.equator-network.org/%20)), life science research (see the [BioSharing Information Resource](https://biosharing.org/)), or the [ARRIVE guidelines](http://www.plosbiology.org/article/info:doi/10.1371/journal.pbio.1000412) for reporting work involving animal research. Where applicable, authors should refer to any relevant reporting standards documents in this form.

If you have any questions, please consult our Journal Policies and/or contact us: [editorial@elifesciences.org](mailto:editorial@elifesciences.org).

**Sample-size estimation**

- You should state whether an appropriate sample size was computed when the study was being designed
- You should state the statistical method of sample size computation and any required assumptions
- If no explicit power analysis was used, you should describe how you decided what sample (replicate) size (number) to use

Please outline where this information can be found within the submission (e.g., sections or figure legends), or explain why this information doesn’t apply to your submission:

Sample sizes were determined to produce enough cells of high quality for sequencing based on pilot experiments, to include biological variables such as sex, and technical replicates to identify contributions of batch effects. These are outlined in the Methods sections: “Mice” and other relevant Methods sections describing each experiment.

Dual RNAscope in situ hybridization-immunohistochemistry and immunofluorescence quantifications sample size and statistics are outlined in the Methods section “Quantifications”.

**Replicates**

- You should report how often each experiment was performed
- You should include a definition of biological versus technical replication
- The data obtained should be provided and sufficient information should be provided to indicate the number of independent biological and/or technical replicates
- If you encountered any outliers, you should describe how these were handled
- Criteria for exclusion/inclusion of data should be clearly stated
- High-throughput sequence data should be uploaded before submission, with a private link for reviewers provided (these are available from both GEO and ArrayExpress)

Please outline where this information can be found within the submission (e.g., sections or figure legends), or explain why this information doesn’t apply to your submission:

Biological and technical replicate information is in the Materials and Methods sections describing each experiment.

Sequencing data is publicly available on GEO at the accession numbers described in: Data & Code Availability

**Statistical reporting**

- Statistical analysis methods should be described and justified
- Raw data should be presented in figures whenever informative to do so (typically when N per group is less than 10)
- For each experiment, you should identify the statistical tests used, exact values of N, definitions of center, methods of multiple test correction, and dispersion and precision measures (e.g., mean, median, SD, SEM, confidence intervals; and, for the major substantive results, a measure of effect size (e.g., Pearson's r, Cohen's d)
- Report exact p-values wherever possible alongside the summary statistics and 95% confidence intervals. These should be reported for all key questions and not only when the p-value is less than 0.05.

Please outline where this information can be found within the submission (e.g., sections or figure legends), or explain why this information doesn’t apply to your submission:

Statistical tests used in single-cell sequencing analyses are provided in the Methods under “Differential Gene Expression & GO Analysis”, and outputs of these tests including raw and adjusted p-values are included for each gene in the Supplementary Tables. Each Table is referenced in the text.

(For large datasets, or papers with a very large number of statistical tests, you may upload a single table file with tests, Ns, etc., with reference to sections in the manuscript.)

**Group allocation**

- Indicate how samples were allocated into experimental groups (in the case of clinical studies, please specify allocation to treatment method); if randomization was used, please also state if restricted randomization was applied
- Indicate if masking was used during group allocation, data collection and/or data analysis

Please outline where this information can be found within the submission (e.g., sections or figure legends), or explain why this information doesn’t apply to your submission:

This does not apply because our analyses did not require ‘treatment’ and ‘control’ groups. Different cell clusters/populations were used to identify differentially expressed genes.

**Additional data files (“source data”)**

- We encourage you to upload relevant additional data files, such as numerical data that are represented as a graph in a figure, or as a summary table
- Where provided, these should be in the most useful format, and they can be uploaded as “Source data” files linked to a main figure or table
- Include model definition files including the full list of parameters used
- Include code used for data analysis (e.g., R, MatLab)
- Avoid stating that data files are “available upon request”

Please indicate the figures or tables for which source data files have been provided:

Supplementary Tables 1-6 provide statistical data presented in the text and figures.

For Figures 3, 4 and 6 we provide source data files containing data of RNAscope and immunofluorescence quantifications.

For Figure 3-figure supplemental 3 and Figure 3-figure supplemental 4 we provide source data files including the gene regulatory networks nodes and edges information

Software packages used for data analysis are referenced in the Materials and Methods, and our code used to implement these software packages will also be made publicly available as described in “Data and Code Availability”.
